# Differential diagnosis of dental fluorosis made by undergraduate dental students

**DOI:** 10.1590/S1679-45082015AO3472

**Published:** 2015

**Authors:** Lilian Rigo, Leodinei Lodi, Raíssa Rigo Garbin

**Affiliations:** 1 Faculdade Meridional, Passo Fundo, RS, Brazil.; 2 Universidade Regional Integrada do Alto Uruguai e das Missões, Erechim, RS, Brazil.; 3 Universidade de Passo Fundo, Passo Fundo, RS, Brazil.

**Keywords:** Fluorosis, dental, Dental enamel hypoplasia, Diagnosis, differential, Students, dental, Tooth abnormalities

## Abstract

**Objective:**

To check knowledge of undergraduate dental students to make diagnosis of dental fluorosis with varying degrees of severity and choose its appropriate treatment.

**Methods:**

Data were collected using a semi-structured questionnaire addressing knowledge of undergraduates based on ten images of mouths presenting enamel changes.

**Results:**

Only three images were correctly diagnosed by most undergraduates; the major difficulty was in establishing dental fluorosis severity degree.

**Conclusion:**

Despite much information about fluorosis conveyed during the Dentistry training, as defined in the course syllabus, a significant part of the students was not able to differentiate it from other lesions; they did not demonstrate expertise as to defining severity of fluorosis and indications for treatment, and could not make the correct diagnosis of enamel surface changes.

## INTRODUCTION

Definitely one of the main clinical concerns is to provide patients the best treatment, including appropriate diagnosis and prognosis. Problems during the correct development of dental structures that determine the shape and function of the dental organ, such as defects or alterations in tooth enamel, may lead to serious impairment that demand from constant monitoring of the affected areas to prosthetic rehabilitation.

In this sense, Passos et al.^(1)^ advocate that enamel defects or alterations are determined by its structure, due to decrease in or loss of translucency in some sites. The authors also suggest that dental tissue structural alterations (enamel) may be a result of local, systemic or hereditary factors, which interfere in tooth mineralization processes.

Regarding oral histology, the odontogenesis process of human dentition (tooth formation) starts during the intrauterine period. Enamel formation (amelogenesis) occurs in three different stages: enamel matrix deposition, calcification (minerals deposition after protein removal), and maturation. Tooth malformations may have many causes, including nutritional factors. Tooth formation may be affected by protein and mineral nutritional deficiencies. Both deciduous and permanent dentitions can be affected and the time of the event is determined by the location of the defect in the dental crown, since the exfoliation and/or eruption processes follow a well-defined chronology.^(2)^

The tissue that covers the tooth crown (enamel) promotes protection and coating. Although this is the most mineralized tissue of the human body, it is extremely sensitive to environmental variations during its formation, which can result in defects. Severe changes in calcium metabolism, low birth weight, traumatic injuries associated with endotracheal intubation and laryngoscopy, trauma and infections in primary teeth, in addition to childhood diseases can be listed as some of the major causes of enamel defects.^(1,2)^

Enamel abnormalities may originate from either quantitative or qualitative defects.^(3)^ Quantitative abnormalities result from a reduction in the amount (thickness) of enamel formed. In other words, there is an insufficient or incomplete formation of the organic matrix, called hypoplasia. A qualitative anomaly occurs when the enamel has normal thickness, but presents changes in its translucency (hypomineralisation), and is called dental fluorosis.^(4)^ This developmental anomaly takes place after prolonged fluorine intake during tooth formation and enamel maturation. It is characterized by increased enamel porosity causing it to appear opaque.^(5)^

One of the most remarkable events of the 20^th^ century regarding oral health was the reduction in the incidence of dental caries, as from the early 1970s. This was considered as a major revolution in health sciences.^(6,7)^ In Brazil, the more plausible explanatory hypothesis to this decline has been the expanded access to fluorinated water and toothpaste, as well as the changes in community dental health programs.^(8)^

Fluorine is said to be the ultimate responsible for this decline. It is added to public water supplies to be provided to the general population. Other fluorine sources are foods, topical applications and fluorinated toothpastes. The use of fluorides over the last decades has led to a decreased incidence of dental caries. However, it also resulted in a higher incidence of fluorosis due to greater exposure of individuals to this microelement associated to an increased intake of fluorinated compounds.^(9)^ Dental caries is currently experiencing a decline in Brazil, mainly due to the widespread use of fluorides in public water supplies and toothpastes. Fluorination of public water supplies has the greatest impact in regions where social conditions are more critical, and where the population does not have access to other means of dental caries protection.^(7,10)^ Despite the benefits of fluorides to prevent tooth decay, it is worth bearing in mind the risks arising from their use, since constant intake of doses higher than those considered safe during the tooth bud formation period may lead to dental fluorosis.^(11,12)^

In a systematic review performed by Ismail and Hasson,^(13)^ the authors analysed evidence of efficacy of fluorine supplements in caries prevention and its association with dental fluorosis. The results showed weak and inconsistent evidence that fluorine supplements prevent tooth decay in primary teeth, but there was strong beneficial evidence for permanent teeth. Mild to moderate fluorosis is a significant side effect. The review included 17 studies on effectiveness of use of fluorine and the possible association with fluorosis. A total of 5294 people were evaluated throughout the five fluorosis studies included in the review.

An increase in diagnosis of dental fluorosis is observed in some Brazilian regions.^(14)^ The Survey on Oral Health Conditions of the Brazilian Population (known as *Projeto SBBrasil*), completed in 2003, showed a prevalence of dental fluorosis in approximately 9% of children aged 12 years, and in 5% of adolescents aged 15 to 19 years. For the 12-year-old group, the highest rates were found in the Southeast and South Regions (roughly 12%) while the lowest rates were in the Central West and Northeast Regions (approximately 4%). In the last *Projeto SBBrasil* survey, carried out in 2010, the prevalence of fluorosis in the 12 year-old group rose to 16.7% - in that, 15.1% were related to very low (10.8%) and low (4.3%) severity levels. Moderate fluorosis was observed in 1.5% of children. The highest prevalence of children with fluorosis was observed in the Southeast Region (19.1%), and the lowest prevalence was found in the North Region (10.4%).^(15)^

During the undergraduate training program in dentistry, information about the clinical features of fluorosis lesions is provided in different subjects,^(16)^ such as oral histology and public health. In order to enable undergraduate students to carry out epidemiological analyses, it is necessary to review the fluorosis levels proposed by Dean, which are currently recommended by the World Health Organization (WHO) epidemiological survey guidelines.^(5,17)^

## OBJECTIVES

To evaluate knowledge of undergraduate dental students when diagnosing and managing cases of dental fluorosis in varied severity degrees.

## METHODS

This is a descriptive study to understand the development of skills and competences of undergraduate dental students to make diagnosis of dental fluorosis, which is a public health problem.

The study population consisted of all 68 students who were taking the Dental Practice subjects, along five semesters (4^th^ to 8^th^) of the undergraduate program in Dentistry at *Faculdade Meridional*. The entry number of students through the admission exam is no more than 20 per semester. However, there sample was 17% smaller due to subjects not attending the interview or not agreeing to participate. Thus, the total sample was 56 undergraduates, distributed into five semesters (Table 1). Out of these, 73.2% (41) were female and 26.8% (15) were male.

**Table 1:** Distribution of undergraduate dental students per semester

Semesters	n (%)
4th	13 (23.2)
5th	9 (16.1)
6th	19 (33.9)
7th	8 (14.3)
8th	7 (12.5)

Total	56 (100)

A semi-structured questionnaire was utilized to collect data and given to 56 undergraduate dental students from the *Faculdade Meridional*, in August 2013. The questionnaire addressed enamel alterations and was given to the participants at the Dentistry School, using slides projected on a one-meter high screen in a dark classroom.

The study was submitted to and approved by the Research Ethics Committee under protocol number 017/2011, CAAE: 0004.0.436.000-11.

The Dean Index was used to determine the presence and absence of dental fluorosis and its degree of severity, ranging from 1 to 5, namely: questionable, very mild, mild, moderate and severe.^(5,17)^

Some images were projected on the screen while the students answered the questions about diagnosis, severity of lesions and treatment. The diagnosis options included normal; incipient caries (white spot lesion); enamel opacity; fluorosis; hypoplasia and “I do not know”. The options for severity of lesions (fluorosis) included the five degrees: questionable, very mild, mild, moderate and severe, as well as “I do not know”. As to treatment, the following options were given: no treatment, non-invasive treatment (for example, plaque control, diet control, prophylaxis and fluorine topical application), invasive treatment (restoration, enamel microabrasion or prosthetic rehabilitation) or “I do not know”.

At the end, the percentages of correct diagnosis of dental fluorosis, severity degree based on the clinical case pictures, and decisions about treatment were assessed.

**Image 1:**
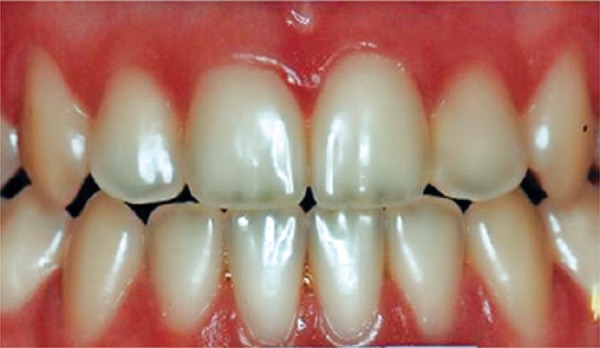
Teeth with no enamel changes

Source: *Projeto SBBrasil 2010: Pesquisa Nacional de Saúde Bucal – Resultados Principais*. http://dab.saude.gov.br/CNSB/sbbrasil/arquivos/projeto_sb2010_relatorio_final

Images showed:

Image 1: The first image shown when applying the questionnaire had normal teeth with no changes. The diagnosis was teeth with healthy enamel.

**Image 2:**
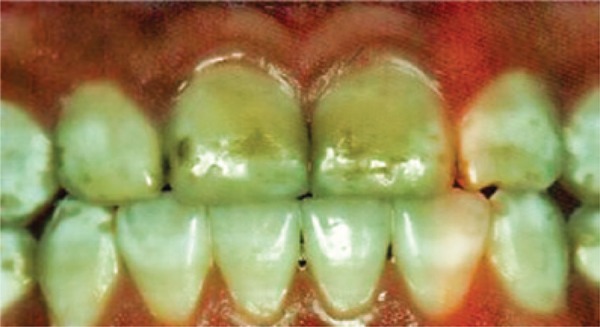
Teeth with severe fluorosis

Source: *Projeto SBBrasil 2010: Pesquisa Nacional de Saúde Bucal – Resultados Principais*. http://dab.saude.gov.br/CNSB/sbbrasil/arquivos/projeto_sb2010_relatorio_final

Image 2: The second image showed fluorosis and the diagnosis was severe dental fluorosis, and invasive treatment would be recommended.

**Image 3:**
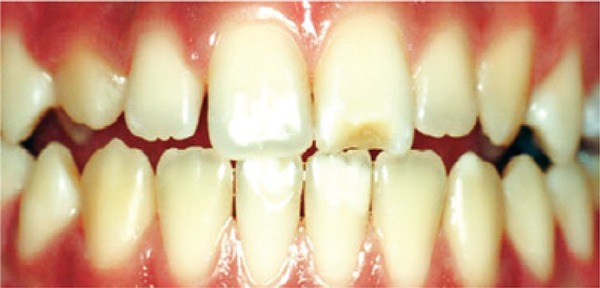
Teeth with enamel hypoplasia

Source: *Projeto SBBrasil 2010: Pesquisa Nacional de Saúde Bucal – Resultados Principais*. http://dab.saude.gov.br/CNSB/sbbrasil/arquivos/projeto_sb2010_relatorio_final

Image 3: The diagnosis of the third image was hypoplasia.

**Image 4:**
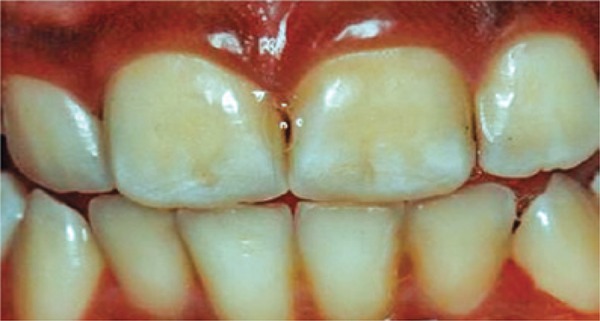
Teeth with mild fluorosis

Source: *Projeto SBBrasil 2010: Pesquisa Nacional de Saúde Bucal – Resultados Principais*. http://dab.saude.gov.br/CNSB/sbbrasil/arquivos/projeto_sb2010_relatorio_final

Image 4: The fourth image should be diagnosed as mild fluorosis, no treatment indicated.

**Image 5:**
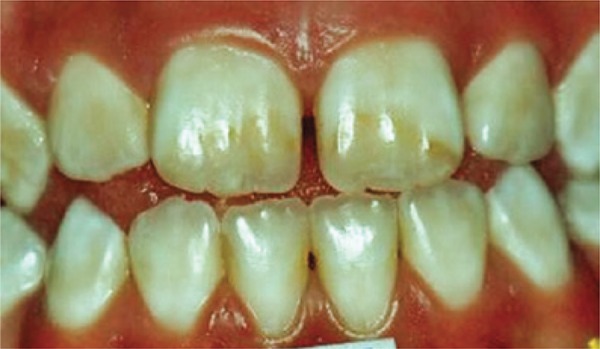
Teeth with moderate fluorosis

Source: *Projeto SBBrasil 2010: Pesquisa Nacional de Saúde Bucal – Resultados Principais*. http://dab.saude.gov.br/CNSB/sbbrasil/arquivos/projeto_sb2010_relatorio_final

Image 5: The fifth picture presented a diagnosis of moderate fluorosis and a non-invasive treatment was recommended.

**Image 6:**
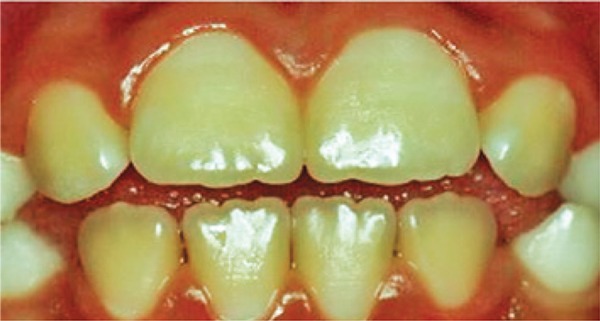
Teeth with very mild fluorosis

Source: *Projeto SBBrasil 2010: Pesquisa Nacional de Saúde Bucal – Resultados Principais*. http://dab.saude.gov.br/CNSB/sbbrasil/arquivos/projeto_sb2010_relatorio_final

Image 6: The diagnosis of the sixth image was very mild fluorosis and no treatment was indicated.

**Image 7:**
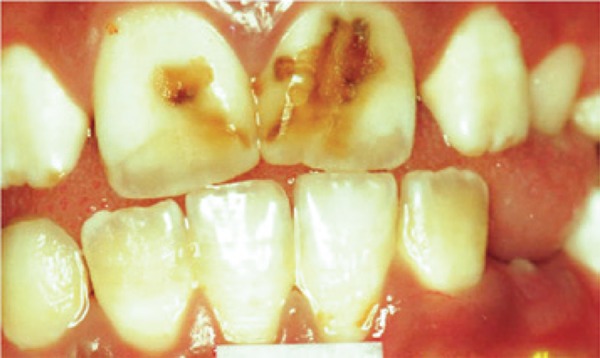
Teeth with severe fluorosis

Source: *Projeto SBBrasil 2010: Pesquisa Nacional de Saúde Bucal – Resultados Principais*. http://dab.saude.gov.br/CNSB/sbbrasil/arquivos/projeto_sb2010_relatorio_final

**Image 8:**
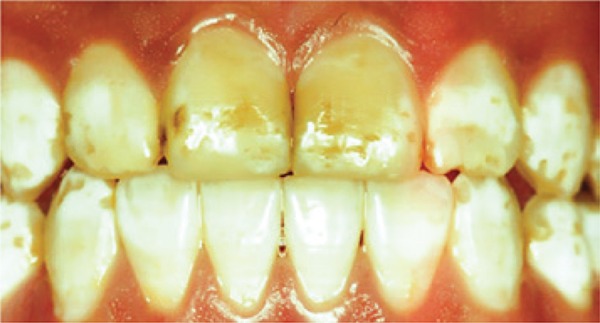
Teeth with severe fluorosis

Source: *Projeto SBBrasil 2010: Pesquisa Nacional de Saúde Bucal – Resultados Principais*. http://dab.saude.gov.br/CNSB/sbbrasil/arquivos/projeto_sb2010_relatorio_final

Image 7 and 8: In the seventh and eighth images, the diagnoses were severe fluorosis and invasive treatment was indicated.

**Image 9:**
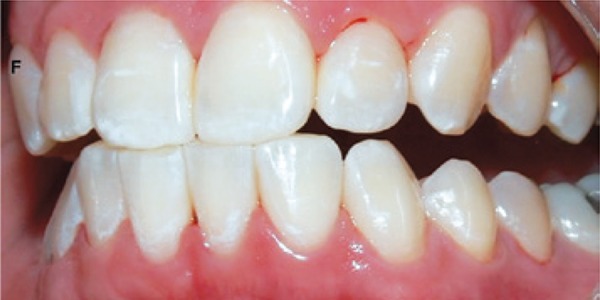
Teeth with very mild fluorosis

Source: *Projeto SBBrasil 2010: Pesquisa Nacional de Saúde Bucal – Resultados Principais*. http://dab.saude.gov.br/CNSB/sbbrasil/arquivos/projeto_sb2010_relatorio_final

Image 9: In the ninth picture, the diagnosis was very mild fluorosis and no indication of treatment.

**Image 10:**
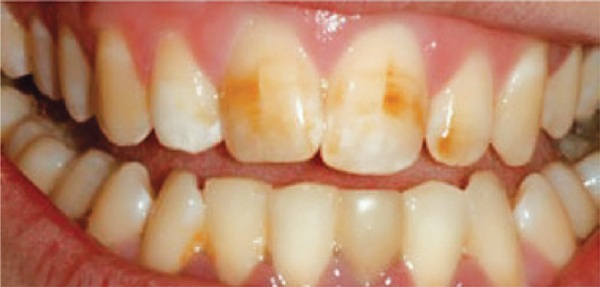
Teeth with moderate fluorosis

Source: *Projeto SBBrasil 2010: Pesquisa Nacional de Saúde Bucal – Resultados Principais*. http://dab.saude.gov.br/CNSB/sbbrasil/arquivos/projeto_sb2010_relatorio_final

Image 10: In the last image, the diagnosis was moderate fluorosis and non-invasive treatment would be recommeded.

## RESULTS

The results of the analysis of answers given by 56 undergraduate students are displayed on table 2.

**Table 2:** Descriptive analysis of ten image-based diagnoses presented to students

Images	Correct diagnosis*		Severity**		Recommended treatment***	
		
		Right answers n (%)		Right answers n (%)		Right answers n (%)
1	Normal	42 (75)	-	-	-	-
2	Fluorosis	38 (67.9)	Severe (5)	6 (10.7)	Invasive	16 (28.6)
3	Hypoplasia	8 (14.3)	-	-	-	-
4	Fluorosis	12 (21.4)	Mild (3)	1 (1.8)	No treatment	2 (3.6)
5	Fluorosis	25 (44.6)	Moderate (4)	3 (5.4)	Non-invasive	10 (17.9)
6	Fluorosis	15 (26.8)	Very mild (2)	0 (0)	No treatment	6 (10.7)
7	Fluorosis	24 (42.8)	Severe (5)	9 (16.1)	Invasive	16 (28.6)
8	Fluorosis	37 (66.1)	Severe (5)	15 (26.8)	Invasive	22 (39.3)
9	Fluorosis	13 (23.2)	Very mild (2)	0 (0)	No treatment	5 (8.9)
10	Fluorosis	26 (46.4)	Moderate (4)	5 (8.9)	Non-invasive	12 (21.4)

* Diagnostic options: normal, incipient caries (white spot lesion), enamel opacity, fluorosis, hypoplasia and “I do not know”; ** Fluorosis severity, according to the Dean Index: (1) questionable; (2) very mild; (3) mild; (4) moderate, (5) severe; *** Non-invasive treatment (*e.g.*, plaque control, diet, prophylaxis and topical fluorine application); and invasive treatment (restoration, enamel microabrasion or prosthetic rehabilitation).

**Figure 1:**
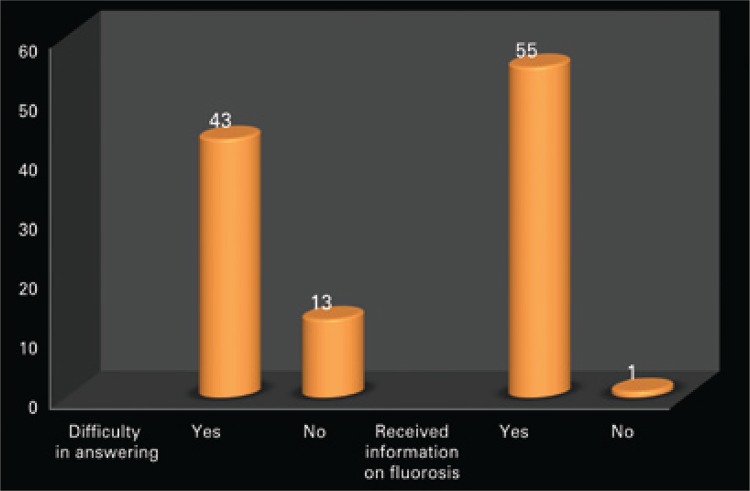
Difficulty in answering the questionnaire and providing information about dental fluorosis

Figure 1 displays the difficulty faced by undergraduate students to answer the questionnaire and provide information on dental fluorosis they had received throughout their training program.

Out of ten diagnoses presented, only three were correctly answered by most undergraduate students: images 1 (healthy enamel - normal), 2 and 8 (severe fluorosis), as shown on table 2. The greatest difficulty was assess severity of fluorosis, which presented low percentage of correct answers. The same was observed in relation to treatment, with few correct answers.

In the first case presented, there were no changes in the enamel and most students made the correct diagnosis (75%). In the second image, a severe case of fluorosis, it was not difficult to make the correct diagnosis (67.9%). However, 39 participants did not know the answer. Only six (10.7%) students correctly answered the degree of severity. Correct treatment indication was only accomplished by 16 (28.6%) students. In the third image, only eight (14.3%) subjects made an accurate diagnosis, *i.e.*, dental hypoplasia; - most undergraduate students interpreted it as early stage dental caries. As to the fourth image, most students (21; 37.5%) chose the diagnosis of enamel opacity, but the correct answer was fluorosis (12; 21.4%), classified as mild (1; 1.8%), and no treatment would be recommended, but only two (3.6%) students answered correctly. The analysis of image five showed a correct diagnosis made by 25 (44.6%) students, whereas the remaining misinterpreted it as enamel opacity and hypoplasia. Only three (5.4%) answered moderate severity correctly. The non-invasive treatment option had only ten (17.9%) right answers. In the sixth image, 15 (26.8%) subjects made the right diagnosis of fluorosis, but none answered correctly in relation to very mild severity. Out of the students who marked the recommended treatment, six (10.7%) got the right option. Twenty-four (42.9%) undergraduates made the correct diagnosis for case 7. However, in relation to the severity, 14 (25%) did not know and 31 (55.4%) did not answer. Only nine (16.1%) chose the correct alternative, *i.e.*, severe dental fluorosis. Concerning therapy, most students (16; 28.6%) got the right answer by choosing invasive treatment. In the eighth picture, 37 (66.1%) subjects made the right diagnosis. When assessing severity level, 15 (26.8%) individuals chose severe. As to treatment indication, most got it correct (22; 39.3%) by choosing the invasive type. When analysing the ninth image, most (22; 39.9%) interpreted it as normal, and only 13 (23.2%) chose fluorosis. The correct severity level - very mild - was not chosen by any student. As to treatment, the majority (30; 3.6%) did not answer, while only five (8.9%) chose the right answer – no treatment recommended. In the last image, the diagnosis of dental fluorosis was correctly made by most students (26; 46.4%). When considering severity, only five (8.9%) chose the correct option - moderate. Non-invasive treatment was only marked correctly by 12 (21.4%) undergraduate students.

After making diagnoses for each image displayed, most students reported difficulties in answering the questionnaire (76%), although they had been informed about dental fluorosis and its severity (Dean Index) during the Dentistry course syllabus (98%), as shown in figure 1.

## DISCUSSION

Dental fluorosis is not a new topic, and its evidence has been more intensively reported and investigated since the fluoridation of public water supplies, in 1974. The Federal Law number 6,050 established mandatory fluorination in municipalities with water treatment units.

Nevertheless, knowledge level of professionals and undergraduate training are causes of concern.

One of the top ten public health measures in the 20^th^ century was fluoridation of water for human consumption,^(7)^ and a significant increase in use of fluorine could be observed as from 1930s, after the advent of fluorinated toothpastes.^(6)^ From then on, the perception of risk factors for dental fluorosis, as well as the decision to use fluorides and their multiple forms to prevent tooth decay, became more relevant. The possible occurrence of fluorosis was one of the reasons that led the Centers for Disease Control and Prevention (CDC) to issue a handbook with recommendations on the use of fluorides^(18)^ in the United States. Brazil also has a manual to guide the professionals.^(19)^

In the present study, the lesions correctly diagnosed by most students were the most severe cases. The opposite was observed as to milder lesions (mild and very mild). In other words, undergraduate students find it more difficult to diagnose dental fluorosis lesions in cases the enamel is not very affected. Making diagnosis of milder cases was more difficult, since there are thin white lines that develop during tooth formation, with no major alterations in tooth colour.

Curiously, in the studies carried out by Levy,^(20)^a group of undergraduates was evaluated before entering the first year of Dental School and, later, at the end of the fourth year, they had a higher perception on many presentations of dental fluorosis, keeping apart enamel opacity and hypoplasia. This change in perception could be explained by exposure to a wider variety of oral conditions during undergraduate training, which led to a minor concern with conditions that are not considered progressive diseases.

In Brazil, in a study with students from the *Universidade de Guarulhos*, São Paulo State,^(21)^ no statistical difference was found when the results of first-semester students were compared with results from the same undergraduates six months later. This suggests that the elapsed time was insufficient to enhance their knowledge on dental fluorosis. According to Narendran et al.,^(22)^ enhanced knowledge about fluorides among healthcare professionals could maximize tooth decay prevention and minimize deleterious effects, such as dental fluorosis.

Baldani et al.^(10)^described that all levels of dental fluorosis were perceived by the groups studied, as opposed to the results of the present investigation, in which students had difficulty in identifying fluorosis, especially in milder cases. This could be observed in the dental hypoplasia case, shown in the third image. Only eight students were able to make an accurate diagnosis, and most diagnosed it as early stage caries.

According to Passos et al.^(1)^ in a more detailed examination, dental surgeons could identify them properly, assessing the etiology and clinical appearance of the alterations. The white spot (initial lesion of caries) has a post-eruption etiology and represents change in tooth enamel due to loss of the structure in the oral environment. Clinically, some changes occur in enamel translucency, which may result in an opaque area, either in the buccal or lingual surfaces. The patient may also present with gingivitis and visible biofilm,^(23)^ differently from hypoplasia, in which there is incomplete or irregular formation of dental enamel.

Some conditions are reported to be essential to appropriate diagnosis of enamel changes and treatment planning. Adequate lighting, drying of teeth and prophylaxis of dental surfaces are factors that contribute to ideal conditions for a clinical examination. These procedures were not performed in this study because the images were shown to students in a classroom setting.^(24)^

According to recent global consensus initiatives for undergraduate dentistry courses, dentists must be competent to apply their knowledge and understand biological, medical and basic sciences, to recognize tooth decay and make decisions about caries prevention and management of individuals and populations. This document presents several competences and is not limited to dental caries, but it also refers to dental erosion and non-erosive dental wear, in addition to other dental hard tissues problems, such as enamel defects.^(25)^

Mastering the differential diagnosis of dental enamel lesions is important in order to collect data correctly, especially in population-based surveys. Dental fluorosis, among the most common enamel defects, is precisely the condition that offers less diagnosis challenge, for it occurs bilaterally and symmetrically. Its diagnosis is easier for being caused by ingestion of fluorides,^(2,12)^ besides its clinical aspect. Other enamel lesions are hypoplasia and hypomineralization. Chronic vitamin deficiencies, particularly vitamin D, are the most common form of enamel hypoplasia. Vitamin A and C deficiencies are also related to enamel hypoplasia. Vitamin A deficiency is known to impair amelogenesis, dentinogenesis and immune function.^(26)^

Image number four led many students to find enamel opacity, misdiagnosing dental fluorosis. When not making the correct diagnosis, the students failed to observe the basic criterion, *i.e.*, fluorotic alterations are symmetrically and bilaterally distributed.^(1,11,12)^ Opacity or hypocalcification do not present structural enamel loss, but rather colour and translucency changes.

Nonetheless, a concern remains - to train skilled professionals to recognize changes and chose the appropriate treatment. As to perception of lesions, patients may not judge the defect as an aesthetic problem - mild fluorosis does not seem to be a concern.^(27)^ It is advisable that dentists should consider patient’s perception to avoid future problems. Whenever any treatment is proposed, patients should also be aware of the limitations, especially in more severe cases.^(1,28)^

From 2010 to 2014, Rigo et al. conducted research in the same city of the present study, and reported high prevalence of dental fluorosis in school-aged children.^(29)^ Severity of fluorosis ranged from very mild to mild, but a high prevalence was associated to female gender and water source.^(30)^ The results demonstrated that more attention should be paid to these communities, establishing how to have access to fluorine, for the population may be exposed to high fluoride content in its various presentations.^(31)^ This fact is quite relevant for the local oral health surveillance and show the need of adequate knowledge on diagnosis of fluorosis by dentists and dental undergraduate students, considering that the incidence of fluorosis was higher than expected in the city of Passo Fundo (RS). Other important studies carried out in Brazil described similar findings.^(32)^

## CONCLUSION

Out of ten abnormalities assessed by undergraduate students, only three were correctly diagnosed. The major difficulty was in determining severity of dental fluorosis.

Despite much information on fluorosis conveyed during the dental training, a significant part of the undergraduates still fail to use it in their clinical practice. The low level of expertise in identifying severity of lesions and indications for treatment demonstrates lack of knowledge to make correct diagnosis of enamel surface alterations.
