# Supplementary material for: Differential diagnosis of dental fluorosis made by undergraduate dental students
Source: Einstein (Sao Paulo). 2015 Oct-Dec;13(4):547–54. doi: 10.1590/S1679-45082015AO3472 (PMC4878629; doi:10.1590/S1679-45082015AO3472)
Supplement: Translation

# Translation: Diagnóstico diferencial de fluorose dentária por discentes de odontologia

**Authors:** Lilian Rigo, Leodinei Lodi, Raíssa Rigo Garbin

**Affiliations:** 1 Faculdade Meridional, Passo Fundo, RS, Brasil.; 2 Universidade Regional Integrada do Alto Uruguai e das Missões, Erechim, RS, Brasil.; 3 Universidade de Passo Fundo, Passo Fundo, RS, Brasil.

**Keywords:** Fluorose dentária, Hipoplasia do esmalte dentário, Diagnóstico diferencial, Estudantes de odontologia, Anormalidades dentárias

## Abstract

**Objetivo:**

Verificar o conhecimento de discentes de um curso de graduação em Odontologia ao diagnosticar casos de fluorose dentária nos diversos graus de severidade, bem como escolher seu tratamento adequado.

**Métodos:**

O levantamento dos dados foi realizado por meio um questionário semiestruturado, que abordou o conhecimento dos acadêmicos sobre as imagens de bocas contendo alterações do esmalte dentário.

**Resultados:**

Apenas três imagens foram diagnosticadas corretamente pela maioria dos acadêmicos; a maior dificuldade foi o diagnóstico da severidade da fluorose dentária.

**Conclusão:**

Apesar das informações sobre fluorose repassadas no decorrer do curso de Odontologia, definidas pelos conteúdos abordados na matriz curricular, expressiva parte dos alunos ainda não soube diferenciá-la de outras lesões, não demonstrando domínio sobre a severidade e as indicações de tratamento, o que indicou desconhecimento no diagnóstico correto das alterações de superfície de esmalte.

## INTRODUÇÃO

Certamente, uma das principais preocupações clínicas é a proposta do melhor tratamento a ser oferecido para o paciente. O tratamento consiste em adequado diagnóstico e determinação de prognóstico, além do tratamento em si. Problemas no desenvolvimento adequado das estruturas dentais, que determinam a forma e a função do órgão dental, no sentido de defeitos ou alterações presentes no esmalte dentário, podem acarretar sérias dificuldades que facultam desde o monitoramento das áreas afetadas, até casos mais graves, que envolvem a reabilitação com próteses.

Neste sentido, Passos et al.^(1)^ preconizam que os defeitos ou alterações do esmalte são determinados pela estrutura, em função da diminuição ou da perda de sua translucidez em alguns locais. Sugerem, ainda, que as alterações na estrutura do tecido dentário (esmalte) podem ser originadas por fatores locais, sistêmicos ou hereditários, e que estes interferem na mineralização dos dentes.

Em se falando de histologia bucal, a odontogênese (formação dentária) da dentição humana começa no período intrauterino. A formação do esmalte (amelogênese) ocorre em três fases distintas: deposição da matriz do esmalte; calcificação (quando os minerais são depositados e as proteínas removidas); e maturação. As causas da malformação dentária são variadas, sendo a nutrição apenas uma delas. O processo de formação do dente pode ser alterado por deficiências nutricionais proteicas e minerais. Tanto a dentição decídua, como a permanente podem ser afetadas, e a época da agressão é determinada pela localização do defeito na coroa dentária, uma vez que os processos de esfoliação e/ou erupção seguem uma cronologia bem definida.^(2)^

O tecido que recobre a coroa dos dentes (esmalte dentário) promove proteção e revestimento. Este é o tecido mais mineralizado do organismo, porém é extremamente sensível às variações do ambiente em sua formação, o que pode resultar em defeitos. Como causa de defeitos em esmalte, destacam-se alterações severas no metabolismo de cálcio, baixo peso ao nascer, danos traumáticos associados à intubação orotraqueal e laringoscopia, traumas e infecções nos dentes decíduos, além das doenças na infância.^(1,2)^

As anomalias do esmalte podem ter origem de defeitos quantitativos ou qualitativos.^(3)^ A anomalia quantitativa decorre da diminuição na quantidade (espessura) de esmalte formado, ou seja, ocorre uma formação deficiente ou incompleta da matriz orgânica, chamada hipoplasia. A anomalia qualitativa ocorre quando o esmalte apresenta espessura normal, mas com alteração na sua translucidez (hipomineralizações), chamada fluorose dentária.^(4)^ A fluorose dentária é uma anomalia do desenvolvimento e ocorre por ingestão prolongada de flúor durante o período de formação dos dentes e da maturação do esmalte. É caracterizada por aumento da porosidade do esmalte, fazendo com que este pareça opaco.^(5)^

A redução da cárie dentária, desde o início da década de 1970, foi um dos fatos mais relevantes do século XX em relação à saúde bucal, sendo considerada uma revolução importante nas ciências da saúde.^(6,7)^ No Brasil, este declínio tem sido acompanhado pela hipótese explicativa mais plausível que consiste na elevação do acesso a água e ao creme dental fluorados, e nas mudanças nos programas de saúde bucal coletiva.^(8)^

O maior responsável por este declínio foi o flúor. Ele é adicionado às águas de abastecimento público, ficando disponível à população. Outras fontes de flúor são os alimentos, as aplicações tópicas e os dentifrícios fluoretados. A utilização dos fluoretos ao longo das últimas décadas diminuiu a incidência da cárie dentária; porém, ocasionou o aumento da fluorose, em virtude de uma exposição maior dos indivíduos a esse microelemento, causada por uma ingestão maior de compostos fluorados.^(9)^ Atualmente, a cárie dentária tem declinado no Brasil, principalmente em razão da ampla utilização de fluoretos nas águas de abastecimento público e nos dentifrícios. A fluoretação das águas de abastecimento público tem o maior impacto em regiões em que as condições sociais são piores e a população não tem acesso a outros meios de proteção.^(7,10)^ Apesar dos benefícios dos fluoretos para a prevenção da cárie dentária, não se pode esquecer dos riscos decorrentes da sua utilização, uma vez que a ingestão constante de doses acima das consideradas seguras, no período de formação do germe dentário, pode desenvolver a fluorose dentária.^(11,12)^

Em um estudo de revisão sistemática realizado por Ismail e Hasson,^(13)^ os autores examinaram evidências da eficácia dos suplementos de flúor na prevenção da cárie e sua associação com a fluorose dental. Os resultados mostraram evidência fraca e inconsistente de que suplementos de flúor impediriam a cárie dentária em dentes decíduos, mas houve forte evidência de benefícios para os dentes permanentes. Fluorose leve a moderada foi um efeito colateral significativo. A revisão incluiu 17 estudos sobre efetividade do uso de flúor e a possível associação com fluorose. Entre os cinco estudos de fluorose incluídos na revisão, 5.294 pessoas foram avaliadas.

Em algumas regiões do Brasil, observa-se um aumento no diagnóstico da fluorose dentária.^(14)^ O Levantamento das Condições de Saúde Bucal da População Brasileira (SBBrasil), concluído em 2003, apontou uma prevalência de fluorose dentária de cerca de 9% em crianças de 12 anos e de 5% em adolescentes de 15 a 19 anos. Para a idade de 12 anos, os maiores índices foram encontrados nas Regiões Sudeste e Sul (em torno de 12%) enquanto que os menores estiveram nas Regiões Centro-Oeste e Nordeste (cerca de 4%). Já no último levantamento do SBBrasil realizado em 2010, a prevalência de fluorose aos 12 anos aumentou para 16,7%, sendo que 15,1% foram representados pelos níveis de severidade muito leve (10,8%) e leve (4,3%). Fluorose moderada foi identificada em 1,5% das crianças. A maior prevalência de crianças com fluorose foi observada na Região Sudeste (19,1%) e o menor valor, na Região Norte (10,4%).^(15)^

Durante a formação profissional do Cirurgião-Dentista, as informações sobre as características clínicas de lesões de fluorose são evidenciadas em diferentes disciplinas,^(16)^ como na histologia bucal e na saúde coletiva. Para capacitar o aluno de graduação a atuar em levantamentos epidemiológicos é necessário revisitar o índice de fluorose dentária proposto por Dean e que atualmente é preconizado nos manuais de levantamento epidemiológico da Organização Mundial da Saúde (OMS).^(5,17)^

## OBJETIVO

Verificar o conhecimento de discentes de um curso de graduação em Odontologia ao diagnosticar casos de fluorose dentária nos diversos graus de severidade, bem como ao realizar seu tratamento.

## MÉTODOS

Estudo descritivo, visando conhecer o desenvolvimento de habilidade e competência dos alunos de graduação do curso de Odontologia para o diagnóstico de fluorose dentária, um problema de saúde pública.

A população do estudo foi constituída por todos os 68 alunos que cursavam as disciplinas de Clínica Odontológica, distribuídos nos cinco semestres (IV ao VIII) do curso de graduação em Odontologia da Faculdade Meridional. O número de alunos por entrada no vestibular é de, no máximo, 20 alunos por semestre. No entanto, houve uma perda de 17% da amostra esperada, em função de o indivíduo não estar no momento da entrevista ou não aceitar participar. Assim, o total de alunos que compôs a amostra foi de 56 alunos, distribuídos em cinco semestres (Tabela 1). Destes, 73,2% (41) eram do sexo feminino e 26,8% (15) do sexo masculino.

**Tabela 1:** Distribuição de alunos por semestres do curso de graduação em Odontologia

Semestres	n (%)
IV	13 (23,2)
V	9 (16,1)
VI	19 (33,9)
VII	8 (14,3)
VIII	7 (12,5)

Total	56 (100)

Como instrumento de coleta de dados, foi utilizado um questionário com questões semiestruturadas, aplicado aos 56 discentes do curso de Odontologia da Faculdade Meridional, no decorrer do mês de agosto de 2013. O questionário abordou alterações próprias de esmalte e foi aplicado na própria instituição, através com o auxílio de slides projetados em uma tela de cerca de 1m de altura, em sala de aula escurecida, para as turmas que participaram da pesquisa.

O trabalho foi submetido e aprovado pelo Comitê de Ética em Pesquisa sob o número de protocolo 017/2011, CAAE: 0004.0.436.000-11.

O índice de Dean foi utilizado para determinar a presença e a ausência de fluorose dentária, e seus graus de gravidade - de 1 a 5, ou seja, questionável, muito leve, leve, moderado e grave.^(5,17)^

Foram projetadas imagens aos alunos que respondiam no questionário suas escolhas de diagnóstico, a severidade das lesões e o tratamento. Dentre as escolhas do diagnóstico, estavam as opções: normal, cárie inicial (mancha branca) opacidade do esmalte, fluorose, hipoplasia e “não sei”. Para as escolhas de severidade das lesões (fluorose), foram apresentadas opções correspondentes aos cinco graus (questionável, muito leve, leve, moderada, severa), além de não sei. Quanto ao tratamento, as opções foram: não indica tratamento, tratamento não invasivo (por exemplo: controle de placa, controle de dieta, profilaxia e aplicação tópica de flúor), tratamento invasivo (por exemplo: restauração dentária, microabrasão do esmalte dentário ou reabilitação com prótese dentária) ou não sei.

Ao final foram avaliados os porcentuais de acertos do diagnóstico de fluorose dentária, do grau de severidade de fluorose baseado nas imagens dos casos clínicos e da decisão do tratamento indicado.

Imagens apresentadas:

Imagem 1: A primeira imagem apresentava dentes normais sem alterações. O diagnóstico dela era de dentes com esmalte dentário sem alterações.

**Imagem 1:** Dentes sem alteração de esmalte

Fonte: Projeto SBBrasil 2010: Pesquisa Nacional de Saúde Bucal – Resultados Principais. http://dab.saude.gov.br/CNSB/sbbrasil/arquivos/projeto_sb2010_relatorio_final

Imagem 2: A segunda imagem apresentava fluorose e o diagnóstico era de fluorose dentária com grau severo cujo tratamento indicado seria o invasivo.

**Imagem 2:** Dentes com fluorose severa

Fonte: Projeto SBBrasil 2010: Pesquisa Nacional de Saúde Bucal – Resultados Principais. http://dab.saude.gov.br/CNSB/sbbrasil/arquivos/projeto_sb2010_relatorio_final

Imagem 3: A terceira imagem apresentava o diagnóstico de hipoplasia.

**Imagem 3:** Dentes com hipoplasia do esmalte

Fonte: Projeto SBBrasil 2010: Pesquisa Nacional de Saúde Bucal – Resultados Principais. http://dab.saude.gov.br/CNSB/sbbrasil/arquivos/projeto_sb2010_relatorio_final

Imagem 4: A quarta imagem tinha como diagnóstico fluorose com severidade leve, sem indicação de tratamento.

**Imagem 4:** Dentes com fluorose leve

Fonte: Projeto SBBrasil 2010: Pesquisa Nacional de Saúde Bucal – Resultados Principais. http://dab.saude.gov.br/CNSB/sbbrasil/arquivos/projeto_sb2010_relatorio_final

Imagem 5: Na quinta imagem, o diagnóstico era de fluorose com severidade moderada; o tratamento indicado era não invasivo.

**Imagem 5:** Dentes com fluorose moderada

Fonte: Projeto SBBrasil 2010: Pesquisa Nacional de Saúde Bucal – Resultados Principais. http://dab.saude.gov.br/CNSB/sbbrasil/arquivos/projeto_sb2010_relatorio_final

Imagem 6: O diagnóstico da sexta imagem era de fluorose com grau de severidade muito leve e sem indicação de tratamento.

**Imagem 6:** Dentes com fluorose muito leve

Fonte: Projeto SBBrasil 2010: Pesquisa Nacional de Saúde Bucal – Resultados Principais. http://dab.saude.gov.br/CNSB/sbbrasil/arquivos/projeto_sb2010_relatorio_final

Imagem 7 e 8: Já na sétima e na oitava imagens, os diagnósticos eram de fluorose com grau severo e tratamento invasivo.

**Imagem 7:** Dentes com fluorose severa

Fonte: Projeto SBBrasil 2010: Pesquisa Nacional de Saúde Bucal – Resultados Principais. http://dab.saude.gov.br/CNSB/sbbrasil/arquivos/projeto_sb2010_relatorio_final

**Imagem 8:** Dentes com fluorose severa

Fonte: Projeto SBBrasil 2010: Pesquisa Nacional de Saúde Bucal – Resultados Principais. http://dab.saude.gov.br/CNSB/sbbrasil/arquivos/projeto_sb2010_relatorio_final

Imagem 9: Na nona imagem, o diagnóstico era de fluorose muito leve e sem tratamento indicado.

**Imagem 9:** Dentes com fluorose muito leve

Fonte: Projeto SBBrasil 2010: Pesquisa Nacional de Saúde Bucal – Resultados Principais. http://dab.saude.gov.br/CNSB/sbbrasil/arquivos/projeto_sb2010_relatorio_final

Imagem 10: Na última imagem, o diagnóstico apresentado era o de fluorose com severidade moderada e indicação de tratamento não invasivo.

**Imagem 10:** Dentes com fluorose moderada

Fonte: Projeto SBBrasil 2010: Pesquisa Nacional de Saúde Bucal – Resultados Principais. http://dab.saude.gov.br/CNSB/sbbrasil/arquivos/projeto_sb2010_relatorio_final

## RESULTADOS

Os resultados da análise das respostas dos 56 discentes foram computados e encontram-se na tabela 2.

**Tabela 2:** Análise descritiva de dez diagnósticos referentes às imagens apresentadas aos discentes

Imagens	Diagnóstico correto*		Grau de severidade**		Tratamento indicado***	
		
		Acertos n (%)		Acertos n (%)		Acertos n (%)
1	Normal	42 (75)	-	-	-	-
2	Fluorose	38 (67,9)	Severa (5)	6 (10,7)	Invasivo	16 (28,6)
3	Hipoplasia	8 (14,3)	-	-	-	-
4	Fluorose	12 (21,4)	Leve (3)	1 (1,8)	Não indica	2 (3,6)
5	Fluorose	25 (44,6)	Moderada (4)	3 (5,4)	Não invasivo	10 (17,9)
6	Fluorose	15 (26,8)	Muito leve (2)	0 (0)	Não indica	6 (10,7)
7	Fluorose	24 (42,8)	Severa (5)	9 (16,1)	Invasivo	16 (28,6)
8	Fluorose	37 (66,1)	Severa (5)	15 (26,8)	Invasivo	22 (39,3)
9	Fluorose	13 (23,2)	Muito leve (2)	0 (0)	Não indica	5 (8,9)
10	Fluorose	26 (46,4)	Moderada (4)	5 (8,9)	Não invasivo	12 (21,4)

* Opções de diagnóstico: normal, cárie inicial (mancha branca), opacidade do esmalte, fluorose, hipoplasia e não sei; ** graus de severidade de fluorose, segundo o Índice de Dean: (1) questionável; (2) muito leve; (3) leve; (4) moderada e (5) severa; *** tratamento não invasivo (por exemplo: controle de placa, dieta, profilaxia e aplicação tópica de flúor); e tratamento invasivo (por exemplo: restauração, microabrasão dental ou reabilitação com prótese).

A figura 1 demonstra a dificuldade dos acadêmicos em responder o questionário e fornecer informações sobre fluorose dentária recebidas durante o período de graduação.

**Figura 1:** Dificuldade em responder ao questionário e informações sobre fluorose dentária

Dos dez diagnósticos apresentados, apenas três foram respondidos corretamente pela maioria representados pelas imagens 1 (esmalte sem lesão – normal), 2 e 8 (fluorose severa), conforme a tabela 2. A maior dificuldade foi acertar a gravidade da fluorose, que teve baixos porcentuais de acertos. O mesmo ocorreu em relação aos tratamentos, havendo poucos acertos.

No primeiro caso apresentado, em que não havia alteração de esmalte, a maioria acertou o diagnóstico de normalidade do esmalte (75%). Na segunda imagem, em que a fluorose apresentou-se severa, não foi dificuldade para a maioria acertar o diagnóstico (67,9%), porém, ao definir a severidade, 39 participantes não souberam responder. Apenas seis (10,7%) alunos acertaram o grau de severidade de fluorose. A indicação correta do tratamento foi feita somente por 16 (28,6%) alunos. Na terceira imagem, apenas oito (14,3%) indivíduos fizeram o diagnóstico correto, que era de hipoplasia dentária − grande parte dos discentes diagnosticou como cárie dentária em fase inicial. Na quarta imagem, grande parte (21; 37,5%) dos alunos assinalou o diagnóstico como opacidade do esmalte, porém o diagnóstico correto seria fluorose (12; 21,4%) com severidade leve (1; 1,8%) e o indicado é não tratar, porém apenas dois (3,6%) alunos responderam corretamente. Analisando a imagem cinco, o diagnóstico foi assinalado corretamente por 25 (44,6%) alunos, porém, os demais confundiram com opacidade do esmalte e hipoplasia. Apenas três (5,4%) acertaram a severidade moderada. A indicação de tratamento não invasivo teve apenas dez (17,9%) acertos. Na sexta imagem, 15 (26,8%) sujeitos acertaram o diagnóstico de fluorose, mas, em relação à severidade muito leve, ninguém acertou. Dos alunos que marcaram o tratamento indicado, seis (10,7%) fizeram escolha certa. No diagnóstico sete, grande parte (24; 42,9%) acertou. No entanto, em relação à severidade, 14 (25%) não sabiam e 31 (55,4%) não responderam. Apenas nove (16,1%) assinalaram a alternativa correta: fluorose dentária severa. Ao escolher o tratamento, boa parte dos alunos (16; 28,6%) acertou assinalando o tratamento invasivo. Na oitava imagem, 37 (66,1%) sujeitos acertaram o diagnóstico. Ao definir a severidade, 15 (26,8%) indivíduos assinalaram severa. Na indicação de tratamento, grande parte (22; 39,3%) acertou, escolhendo o tratamento invasivo. Ao analisar a nona imagem, boa parte (22; 39,9%) indicou como sem alteração, e apenas 13 (23,2%) marcaram fluorose. A severidade correta, muito leve, não foi assinalada por nenhum aluno. Na indicação do tratamento, a maioria (30; 3,6%) não respondeu, enquanto apenas cinco (8,9%) acertaram − não era indicado tratar. Na última imagem, o diagnóstico de fluorose dentária estava correto por boa parte dos alunos (26; 46,4%). Ao analisar a severidade, apenas cinco (8,9%) acertaram ser a moderada. O tratamento não invasivo foi assinalado corretamente por somente 12 (21,4%) alunos.

Ao final dos diagnósticos feitos para cada imagem apresentada, a maioria dos discentes relatou ter dificuldades em responder ao questionário (76%), apesar de terem recebido informações sobre fluorose dentária e sua severidade (Índice de Dean) durante o curso de Odontologia (98%), como demonstrado na figura 1.

## DISCUSSÃO

A fluorose dentária não é um tema novo, e sua evidência tem sido relatada e pesquisada mais intensamente, desde o início da fluoretação das águas de abastecimento público em 1974, com a lei federal 6.050, que determina a obrigatoriedade da fluoretação dos municípios com estações de tratamento de água. No entanto, o nível de conhecimento dos profissionais e o estudo durante a formação profissional nos cursos de graduação são motivos de preocupação.

No mundo, uma entre as dez medidas mais importantes de saúde pública do século 20 foi a fluoretação das águas de consumo humano,^(7)^ e o significativo aumento do uso de flúor pôde ser observado a partir dos anos de 1930 com a fluoretação dos dentifrícios.^(6)^ A partir disso, a percepção dos fatores de risco para fluorose dentária, assim como a decisão para o uso de fluoretos e suas múltiplas formas para a prevenção de cárie dental, passa a ganhar mais importância atualmente. Essa possibilidade de ocorrência de fluorose foi um dos motivos que levou o *Centers for Disease Control and Prevention* (CDC) a divulgar um manual com recomendações no uso de fluoretos^(18)^ nos Estados Unidos; no Brasil, um manual também orienta os profissionais.^(19)^

No presente estudo, as lesões diagnosticadas corretamente pela maioria dos alunos foram as lesões de fluorose, cuja severidade é mais grave. O contrário ocorreu com as lesões de severidade mais branda (leve e muito leve). Os discentes têm maiores dificuldades em diagnosticar as lesões de fluorose dentária nos casos em que o esmalte está pouco comprometido. Percebe-se a dificuldade em diagnosticar os casos mais brandos, pelo fato de apresentarem finas linhas brancas que acompanham a formação dentária, sem mudanças exageradas na cor dos dentes.

Nos estudos de Levy,^(20)^ curiosamente, um mesmo grupo de estudantes de Odontologia avaliado antes de entrar no primeiro ano e, novamente, no final de seu quarto ano tiveram maior percepção sobre muitas apresentações de fluorose dentária, isolando as opacidades e hipoplasias. Essa mudança de percepção poderia ser explicada devido à exposição a uma grande variedade de condições bucais durante a formação profissional, que levou a uma menor preocupação com as condições que não são doenças progressivas.

Já no Brasil, em um estudo com alunos da Universidade de Guarulhos, no estado de São Paulo (SP),^(21)^ nenhuma diferença estatística foi encontrada quando os resultados dos alunos do primeiro semestre foram comparados com os apresentados pelos mesmos discentes, seis meses depois. Isso sugere que o tempo decorrido foi insuficiente para aumento no nível de conhecimento sobre a fluorose dentária. De acordo com Narendran et al.,^(22)^ uma melhoria do conhecimento de fluoretos entre profissionais de saúde pode maximizar a prevenção da cárie dentária e minimizar os efeitos deletérios como a fluorose dentária.

Baldani et al.^(10)^ descrevem que todos os níveis de fluorose dentária foram percebidos pelos grupos estudados, contradizendo os resultados encontrados neste estudo, no qual os alunos tiveram dificuldade em identificar fluorose, principalmente nos graus mais leves. Isso ocorreu, por exemplo, no caso da imagem de hipoplasia dentária, a terceira imagem apresentada, para a qual apenas oito discentes fizeram o diagnóstico correto, enquanto que a maioria diagnosticou como cárie dentária inicial.

Segundo Passos et al.,^(1)^ em um exame mais minucioso, o dentista poderia identificá-las corretamente, analisando a etiologia e a aparência clínica das alterações. A mancha branca (lesão inicial de cárie) é uma alteração no esmalte dentário, devido às perdas da estrutura para o meio bucal, sendo sua etiologia pós-eruptiva. Clinicamente, há mudança de translucidez do esmalte, aparecendo uma área opaca, que pode estar nas áreas vestibular e lingual. O paciente pode apresentar gengivite e biofilme dentário visível,^(23)^ diferentemente da hipoplasia, que apresenta formação incompleta ou irregular do esmalte dentário.

Existem algumas condições que são relatadas como fundamentais para o diagnóstico das alterações de esmalte e para o planejamento do tratamento adequado. Dentre elas, são consideradas como condições ideais para realização do exame clínico a iluminação adequada, a profilaxia das superfícies e a secagem dos dentes. Esses procedimentos não ocorreram no presente estudo, pois se tratavam de imagens projetadas em sala de aula para os alunos.^(24)^

De acordo com recentes iniciativas de consenso mundial em graduação em Odontologia, um dentista deve ser competente para aplicar os conhecimentos e ter compreensão das áreas biológicas, médicas, ciências básicas, para reconhecer a cárie dentária e tomar decisões sobre sua prevenção e “gestão” para indivíduos e populações. Este documento apresenta várias competências e não se limita apenas à cárie dentária, mas refere-se também à erosão dental e ao desgaste não erosivo, além de outros problemas dentários dos tecidos duros, como os defeitos de esmalte.^(25)^

Dominar o diagnóstico diferencial das lesões de esmalte dentário é importante, no sentido de colher o dado da forma correta, principalmente em levantamentos de base populacional. É justamente a fluorose dentária, dentre os defeitos do esmalte mais comuns, a que apresenta a maior facilidade no diagnóstico, por ocorrer bilateralmente e de forma simétrica, e ainda ter como etiologia a ingestão de fluoretos, que, juntamente do aspecto clínico, facilita o diagnóstico.^(2,12)^ Outras lesões de esmalte são as hipoplasias e hipomineralizações. A deficiência crônica de vitaminas, particularmente da vitamina D, é a forma mais comum de hipoplasia de esmalte. As vitaminas A e C também estão relacionadas com a hipoplasia de esmalte. A deficiência de vitamina A é conhecida por alterar a amelogênese, a dentinogênese e a função imunonológica.^(26)^

Na imagem de número quatro, na qual muitos alunos indicaram opacidade do esmalte, não acertando o diagnóstico de fluorose, verifica-se que, ao errar o diagnóstico correto, os alunos não observaram o critério básico de que as alterações de fluorose são distribuídas simetricamente e bilateralmente.^(1,11,12)^ Nem a opacidade e nem a hipocalcificação apresenta perda estrutural do esmalte, mas sim mudança em sua cor e translucidez.

Uma preocupação permanece: a de formar profissionais capacitados tanto para o reconhecimento das alterações, assim como a indicação do tratamento adequado. Quanto à percepção das lesões, o paciente pode não julgar o defeito como problema estético − a fluorose leve parece não ser uma preocupação.^(27)^ Aconselha-se ao cirurgião-dentista que considere a percepção do paciente, no sentido de evitar transtornos futuros. Quando proposto o tratamento, o paciente também deve estar ciente das limitações, principalmente nos casos mais severos.^(1,28)^

De acordo com Rigo et al., no período de 2010 a 2014, no mesmo município da presente pesquisa, houve prevalência elevada de fluorose dentária nos escolares.^(29)^ A severidade de fluorose encontrada foi leve e muito leve, porém a alta prevalência esteve associada com o sexo feminino e a fonte de água.^(30)^ Os resultados indicaram a necessidade de atenção para estas comunidades, estabelecendo as formas de acesso ao flúor, pois a população pode estar tendo um consumo elevado do fluoreto em suas várias formas de apresentação.^(31)^ Esse fato é importantíssimo para a vigilância em saúde bucal da localidade e para um conhecimento adequado sobre o diagnóstico da fluorose pelos cirurgiões-dentistas e acadêmicos de Odontologia, uma vez que frequências de fluorose mais altas que as esperadas têm sido encontradas no município de Passo Fundo (RS), assim como em outros estudos importantes no Brasil.^(32)^

## CONCLUSÃO

Das dez alterações analisadas pelos alunos, apenas três foram diagnosticadas corretamente pela maioria dos acadêmicos. A maior dificuldade foi em relação ao grau de severidade da fluorose dentária.

Apesar da grande quantidade de informações sobre fluorose repassadas no decorrer da matriz curricular, uma expressiva parte dos alunos ainda não sabe empregá-la em sua prática clínica e não tem domínio da severidade e das indicações de tratamento, demonstrando desconhecimento no diagnóstico correto das alterações de superfície de esmalte.
